# Complexities of competency and informed consent as applied to individuals with symptoms of Anosognosia

**DOI:** 10.1017/S109285292400227X

**Published:** 2025-03-21

**Authors:** Nina M. Labovich

**Keywords:** Anosognosia, schizophrenia, informed consent, law, courts

## Abstract

Anosognosia, commonly understood as a lack of insight, renders individuals with schizophrenia and schizoaffective disorder unable to understand that they are living with a disease, often resulting in a refusal to accept treatment. Typically, to impose involuntary commitment in an effort to obtain treatment, an individual must be a danger to others or themselves. Even if involuntary commitment is imposed, however, an individual may remain competent to refuse *medication*—despite symptoms of anosognosia and an inability to understand that they are ill. This article examines the existing legal theories of competency and informed consent and proposes a statutory definition of competency that encompasses the specific needs of people with anosognosia, while considering the significant interests at stake when taking away an individual’s right to choose or refuse treatment.

## Introduction

“As if the symptoms of schizophrenia were not devastating enough in themselves, nature has added a cruel joke, a seemingly valueless yet powerful barrier between the sufferer and professionals reaching out to help. The cruel joke is called anosognosia.”^1^ ^(pp26–27,292–295)^

Imagine you are a sophomore in college. You have been diagnosed with a serious mental illness and take medication to manage your symptoms. At first, you check in with your family and keep them updated on your condition, therapy, and medications. Several successful months on medication later, however, you know that you can control your own symptoms and medication. You stop taking your medication because you are fine and you do not have a mental illness—how could your family or doctor know more than you about *your* condition? Your family visits and begs you to get back on your medication. You refuse. Your doctor finds that you have the medical capacity to refuse treatment because you can understand the risks and benefits of the treatment, even though you cannot see that you are sick. You refuse medication, and eventually, you drop out of college due to your lack of control over your symptoms. You are not violent, so the state does not impose involuntary commitment. But you eventually become homeless, disconnected from family, and have worsening symptoms due to lack of medication.

This representative story demonstrates how anosognosia may impact an individual living with mental illness. Anosognosia may render a person unable to see that they are sick, which can snowball into refusing medication, worsening symptoms, and, unfortunately, homelessness or incarceration.^2^ Separate from the devastating personal implications of anosognosia, the symptom may have critical legal implications as well.

Although medication may be, arguably, the best option for a patient with schizophrenia and anosognosia, prior to administering medication, the doctor must explain the pros and cons of accepting the treatment and ask for the patient’s informed consent.^3^ ^(§1)^ Informed consent occurs when a patient either accepts or declines treatment, following a doctor’s explanation of risks involved in the procedure.^4^ For an individual to give informed consent, a doctor must first find that the person can make medical decisions.^5^ ^(§4.1)^

If there is a dispute concerning a person’s capacity to make medical decisions, a judge may be required to make a legal competency determination, or in other words, determine whether the individual is considered competent to refuse treatment under the governing state statute. Medical capacity and legal competency findings have some overlap, as both require a careful analysis of an individual’s ability to make decisions, but the focus of this article is on the legal competency analysis. Although it may seem logical that a person who cannot see his or her illness cannot give informed consent, the law is inconsistent—in some jurisdictions, denial of illness warrants incompetency, but in others, it does not.

This article attempts to develop (or at minimum, articulate the challenges of developing) a legal solution, via a model definition of “competent,” to the question of competency for individuals living with anosognosia. As is obvious from the history of mental health treatment in the United States—ranging from overinstitutionalization to wholly ignoring people living with mental illness—no solution is perfect and the path forward is muddy; however, at a bare minimum, the law should evolve with the science.

## Informed consent and theories of competency

The doctrine of informed consent is grounded in an essential premise of the U.S. legal tradition—all individuals have the right to make their own choices.^6^ ^(p1104),^^7^ ^(p780)^ As a result, in most circumstances, a doctor cannot ignore the individual’s choice, even if the doctor thinks it is unwise.^6^ ^(p1104)^ There are narrow exceptions to this rule, such as in an emergency or, as is most relevant here, when a judge finds a patient legally incompetent to consent to treatment.^8^ ^(p919)^ This article only considers competency to refuse treatment, not, for example, competency to stand trial; there are a variety of types of competency, each requiring different elements and legal tests.

Generally, adults are considered competent to make their own treatment decisions under the law.^5^ ^(§4.1)^ If a patient’s legal competency is questioned, a physician must assess whether the patient has the medical capabilities to make decisions, often referred to as determining a patient’s capacity.^9^ ^(pp743-744),^^10^ ^(pp348-349)^ Note that the medical capacity test is different from legal competency, although in crafting definitions of competency or incompetency, theorists and legislatures will consider the elements and extent of medical capacity required to be considered competent under their law.^10^ ^(pp348-349)^ With respect to medical capacity, doctors generally base that determination on the patient’s ability to do the following: understand the situation, communicate a choice to the physician, appreciate the potential outcomes, and reason or rationalize (in other words, weigh the pros and cons) generally.^11^

When patients dispute a physician’s determination of medical capacity, a judge may be required to make a legal competency determination; there is, however, no general consensus among approaches to competency determinations and definitions, with statutes varying across the country, state by state.^10^ ^(pp348-351)^ In any event, upon a finding of incompetence, an individual will not be able to make treatment decisions directly, requiring a surrogate to make decisions on the patient’s behalf.^12^ ^(pp104–105)^

At a minimum, experts have identified four prominent legal theories of competency, although there are certainly others.

1. The patient can articulate a choice.

The first theory, the mere “ability to communicate a choice,” requires the least rigorous review of the patient’s capabilities: when a patient can communicate a decision, the patient is deemed competent.^10^ ^(p352)^ In effect, only patients in comas or vegetative states would be deemed incompetent under this theory.^10^ ^(pp352-353)^ Although this theory of competency arguably may be the most dependable theory (because it is subject to the least amount of interpretation), it is insufficient to establish competency alone.^9^ ^(pp744-745),^ ^10^ ^(p353)^ Rather, the ability to communicate a choice often is one component of existing theories.^10^ ^(p353)^

2. The patient can understand—but cannot appreciate—the information presented.

A second theory—often applied in the United States—tests whether a patient can understand the information presented.^13^ ^(p952),^ ^10^ ^(p353)^ In jurisdictions that use this theory, courts require an individual to “comprehend the concepts involved,” but the patient need not *fully appreciate* the situation.^10^ ^(pp353–354)^ As applied, a patient would solely need to understand what a disease or symptom is, but not appreciate that the symptom or disease is *applicable* to the patient themself.^10^ ^(pp353–354)^ For example, Idaho adopts the “understanding” theory, defining “[l]acks capacity to make informed decisions” as the “inability… to achieve a rudimentary understanding of the purpose, nature, and possible risks and benefits of a decision.”^14^

3. The patient can understand *and* appreciate the information.

A third, more stringent theory, requires the patient to both understand the information presented and to understand that the information is applicable to the patient.^10^ ^(pp355–357),^ ^13^ ^(p955)^ In jurisdictions using this theory, to be deemed competent the patient must understand the information and appreciate the fact that the information applies to the patient and may carry certain consequences for the patient.^10^ ^(pp355–357)^ State statutes incorporating this theory may vary; for example, in Alaska, the relevant statute explicitly requires patients to appreciate that they have an impairment to be deemed competent,^15^ whereas in Tennessee, the statute merely requires that patients are “able to understand and appreciate the nature and consequences” of their treatment decisions.^16^

4. The patient understands, appreciates, and makes a rational decision.

Finally, some theories of competency require that a patient can understand and appreciate the information provided, *and* make a rational decision concerning treatment.^13^ ^(pp956-957),^ ^10^ ^(pp357–358)^ This approach examines whether the patient is capable of logically deciding on treatment by considering all of the information offered and weighing the pros and cons of the proposed treatment.^10^ ^(pp357-358)^ As one example, in Alabama, individuals are considered “incapacitated person[s]” when they become impaired such that they “lack[] sufficient understanding or capacity to make or communicate responsible decisions.”^17^

## Applying legal theories of competency to people with anosognosia

Scholars, courts, and legal practitioners disagree about the competency of individuals with schizophrenia and symptoms of or like anosognosia. Due to the patchwork legal framework in the United States, and the reliance upon individual judges and triers of fact to apply the legal standards in a variety of circumstances, it is difficult to cleanly silo court decisions and legal theories into one category or another. The following section summarizes three approaches, over a broad time period, and is meant to be illustrative, not exhaustive.

### An individual with a lack of insight is competent

In 1994, in *In re Virgil D.*, the Supreme Court of Wisconsin held that an individual with schizophrenia who was unable to recognize his illness was competent to refuse antipsychotic medication, reversing the decision of the Court of Appeals of Wisconsin that had found the patient’s inability to understand his illness warranted incompetency under state law.^18(p895)^ Applying the plain language of the statute, which merely required a patient to understand the effects, benefits, and risks of a particular treatment, the Supreme Court of Wisconsin concluded that it was of no moment that the patient could not understand or accept his sickness; he merely needed to understand the particulars of the *treatment.* ^18^ ^(pp898–900)^

There are notable benefits to finding an individual with anosognosia competent. For one, given the misunderstanding of individuals living with mental illnesses, patients that repeatedly deny their illness may not be suffering from anosognosia, but instead could be avoiding the stigma of being diagnosed with mental illness.^13^ ^(p990)^ In addition, finding patients incompetent any time they denied a supposed illness could have severe implications. In theory, and at its most extreme extension, permitting denial of a mental illness to warrant a finding of incompetency could lead to individuals being committed wrongfully for fringe or unpopular ideas.^13^ ^(p992)^ Finally, a patient’s lack of insight may not rise to the level of anosognosia or be sufficiently pervasive to warrant a finding of incompetency. Without a documented history of patients’ behavior, it may be difficult to determine whether a patient is in denial or living with anosognosia.^19^ ^(p49)^

### If a patient denies obvious symptoms or holds erroneous beliefs, they are incompetent

According to one prominent theorist, anosognosia, coupled with absurd beliefs or denials of verifiable symptoms of schizophrenia, warrants a finding of incompetency.^20^ ^(pp181–182)^ For example, when an individual denies an objectively apparent symptom of schizophrenia (*eg.*, insomnia), a finding of incompetency would be justified because denial of a quantifiable symptom demonstrates that the individual’s mental processing is so broken down that such an individual is incompetent.^13^ ^(p991)^ Likewise, a patient who denies their illness because they believe the symptoms of schizophrenia arise as a result of an infestation of evil spirits (as opposed to a brain disorder) should be found incompetent.^13^ ^(p991)^ In either circumstance, there is clear evidence that the patient has delusions and symptoms of schizophrenia; coupled with a lack of insight, there is cause for a finding of incompetency.^13^ ^(pp991–992)^ When a patient’s denial of illness is caused by delusions or the patient denies visible symptoms, there may be less cause for concern that a court is wrongfully taking away a patient’s right to refuse treatment. Requiring gross denial of symptoms or plainly false beliefs *and* denial of objectively apparent symptoms of the illness may help mitigate concerns that a patient is in denial, misdiagnosed with a psychiatric condition, or wrongfully persecuted for his or her beliefs.^13^ ^(pp991–992)^ Denial of an apparent symptom demonstrates that a patient cannot understand a diagnosis or appreciate its impact—requirements of many informed consent statutes. Separately, this view may encourage patients and doctors to thoroughly explore the individual’s reasoning.^21^ ^(p973)^

### A patient with anosognosia is incompetent

In 2017, in *People v. D.A.*, a California appellate court held that an individual with schizophrenia and severe anosognosia was incompetent and could not give informed consent.^22^ ^(p*1–2)^ As a result, the court upheld the involuntary administration of antipsychotic medication, emphasizing testimony from the patient’s doctor, who stated that the patient did not understand he had schizophrenia and refused to take his medication. The doctor testified that as a result of the lack of medication, the individual had experienced worsening symptoms and felt persecuted by those trying to treat him.^22^ ^(p*2–4)^ The court considered the patient’s ignorance of his symptoms and diagnosis and found that he was unable to weigh the pros and cons of medication and could not productively participate in his treatment decision due to his lack of insight.

The doctrine of informed consent aims to protect patient free will.^23^ ^(p346)^ To effectuate autonomy, competency tests ensure that patients who are able to make their own decisions are, in fact, permitted to have that choice.^23^ ^(p348)^ Scientific evidence arguably supports a finding of incompetency on the basis of anosognosia, as it indicates anosognosia interferes with the ability of a patient to make decisions about their treatment.^12^ ^(pp103–104)^ Psychiatric conditions directly affect parts of the brain that allow individuals to think, make decisions rationally, and understand the likelihood of events happening in the future.^24^ ^(p804)^ Arguably, without proper insight into their conditions, individuals cannot make a free choice.^24^ ^(p805)^ Instead, the choice has been made by the disease because it has infiltrated their decision-making capacity. Allowing patients with poor insight to decline medication does not necessarily affect their free choice, which is the goal of informed consent, because the disease dictates the choice.^24^ ^(p805)^ A finding of incompetency on the basis of anosognosia, then, may help doctors best determine and effectuate a patient’s autonomous choice.

Separately, finding patients incompetent on the basis of anosognosia may help retain patients’ ability to choose in the long term.^24^ ^(p804)^ When patients with anosognosia refuse treatment in the present, they may be making a decision *never* to treat because their lack of insight will likely worsen without treatment over time. With treatment, in contrast, symptoms of anosognosia may lessen, potentially allowing patients to *gain* insight over time.^24^ ^(p803)^ Some argue that if the goal of informed consent is to effectuate autonomy, ensuring patients’ long-term autonomy is best accomplished by finding patients with anosognosia incompetent in the present.^24^ ^(pp804–805)^

## Charting a compassionate path forward: documented anosognosia warrants legal incompetency

### Appreciation of illness is fundamental to the goals of informed consent and necessary for a finding of competency

Effectuating and respecting an individual’s autonomous choice is, ultimately, the goal of informed consent. As explained above, schizophrenia may have direct impacts on the parts of the brain responsible for an individual’s decision-making. Anosognosia impacts an individual’s ability to think rationally and directly affects the brain’s ability to accurately process information. If an illness, like schizophrenia, or symptom, like anosognosia, directly affects the part of the brain that contributes to a person’s ability to freely make choices, the illness or symptom must be considered in the competency determination.^24^ ^(pp804–805)^ Under those circumstances, it cannot be said that the patient is actually acting with autonomy, cutting against a central tenet of informed consent.

Although traditional notions of informed consent focus on a patient’s current state of mind, when a patient’s decision in the present will effectively serve as the only meaningful opportunity to decide, considering future implications of the decision are warranted. Accordingly, if the goal of informed consent is to effectuate a patient choice, informed consent must consider whether a patient can appreciate the illness, particularly where an illness can obfuscate a patient’s actual decision-making.^24^ ^(pp804–805)^

### A balance between patient autonomy and modern science

Despite some jurisdictions already requiring patients to “appreciate” their illness to exercise their right to refuse treatment, statutory language varies. For example, in Alaska, the statutory definition of competency explicitly requires, *inter alia*, patients to appreciate that they have an impairment.^15^ In contrast, Tennessee’s statute is less specific.^16^

Alaska’s statutory language provides the clarity necessary to ensure that anosognosia is considered in a judge’s competency determination, and provides a starting point for model statutory language. This article, however, contends that the statutory language must go further to both protect individuals and reflect modern science. Concrete statutory language would protect patient autonomy, by ensuring those who have insight into their condition but, for their own reasons, do not want to undergo treatment, are found competent to refuse treatment. Separately, detailed language would ensure that only those who have medically documented anosognosia and not mere denial are found incompetent. Explicit language safeguards against judges reading their own beliefs about denial into their statutory interpretation. And, most importantly, tailored language ensures that the statute reflects the best science available to test for anosognosia. In sum, the article proposes a model statute incorporating Alaska’s language and includes additional components that aim to protect an individual’s right to choose treatment. This proposed statute moves the conversation toward a humane approach to effectuating autonomy for people with anosognosia.

“Competent” means that the person:

- Has the ability to assimilate relevant facts and to appreciate and understand their situation with regard to those facts^15^;
- Appreciates that they have a mental illness or impairment, if the evidence so indicates^15^
  - A person’s denial of his or her mental illness is evidence that the person lacks the capability to make treatment decisions when the person’s denial is a result of anosognosia, a significant deficit in insight.^19^ ^(p49)^ Denial is evidence of anosognosia when it has lasted over a period of at least six months, the denial does not change even if the individual is presented with evidence, and the individual offers alternative absurd explanations to persuade others that he or she does not have an illness.^19^ ^(p49)^
    - The lack of insight must be documented during a period of at least six months, and a doctor must be willing to testify to the patient’s lack of insight as a symptom of his or her disease, not mere denial.^19^ ^(p49)^
- If a person is found incompetent under Section b, he or she may be administered antipsychotic medication involuntarily, if a doctor has determined it is appropriate.^22^ ^(p*3)^ When and if the person obtains insight into his or her medical condition (or in one year, whichever time period is sooner) the person’s capacity must be reassessed by a medical professional and the person’s competency must be reassessed by a judge.

## Author Biography

Nina M. Labovich is an attorney. Previously, she was a judicial law clerk at the US District Court for the District of Massachusetts. She is a graduate of Boston College Law School and Wesleyan University. This article is an abbreviated version of her Note, published in the Boston College Law Review in 2021, during her third year of law school. Opinions expressed in this article are the author’s own and do not reflect the views of her employer.
